# Occupation, displacement, and violence in the West Bank: A retrospective analysis of data from 2014–2024

**DOI:** 10.1371/journal.pgph.0004829

**Published:** 2025-06-25

**Authors:** Sarah Aly, Fatima Mossolem, Ayeh Khalil, Tushara Surapaneni, Abd Al-Rahman Traboulsi, Waleed Aldadah, Eleanor Reid, Shakoor Hajat

**Affiliations:** 1 Department of Emergency Medicine, Yale University, New Haven, Connecticut, United States of America; 2 Health in Humanitarian Crises Centre, London School of Hygiene and Tropical Medicine, London, United Kingdom; 3 Rowan School of Osteopathic Medicine, Stratford, New Jersey, United States of America; 4 Columbia University Mailman School of Public Health, New York, New York, United States of America; 5 Department of Emergency Medicine, Massachusetts General Hospital Brigham, Boston, Massachussets, United States of America; 6 Department of Emergency Medicine, HCA Florida Kendall Hospital, Miami, Florida, United States of America; Bielefeld University, GERMANY

## Abstract

Israeli settlement expansion in the West Bank has intensified violence in the occupied Palestinian territories (oPt). This violence escalated after attacks by Hamas on Israel on 7 October 2023 and the subsequent military campaign in Gaza. This study examines the possible impacts of military and land occupation on displacement, injuries, and deaths in the West Bank. This cross-sectional observational study analyses casualties and displacement data in the West Bank and Israel from May 1, 2014, to June 30, 2024. Sources include the United Nations Office of Coordination of Humanitarian Affairs, Statista, and the Palestinian Central Bureau of Statistics. Death and injury rates per 100,000 person-years were calculated and compared across populations. Interrupted time-series analysis compared observed Palestinian deaths, injuries, and displacement to expected levels since October 2023. Chi-square analysis examined demolition patterns by West Bank area. GIS mapping methods visualized spatial variations in casualties and demolitions. Death and injury rates were substantially higher for Palestinians than Israelis: RR = 5.72 (95% CI 2.38, 13.75; *p* < 0.001) for deaths and RR = 16.47 (6.86, 39.56; *p* < 0.001) for injuries. Refugee camps had increased death rates: IRR = 7.91 (5.26, 11.89; *p* < 0.0001) compared to non-refugee camp populations. Since October 2023, West Bank deaths were 25% higher than expected: RR = 1.25 (1.15, 1.36; *p* < 0.0001) and displacement 17% higher: RR = 1.17 (1.12, 1.21; *p* < 0.0001). Nablus and Jenin recorded the highest fatalities. Jabal al-Mukkabir in East Jerusalem experienced the highest number of demolitions. Our study confirms a significant disparity in rates of conflict-related traumatic injuries and deaths between Palestinians and Israelis. The findings emphasize the need to limit military force against civilians, to hold the Israeli government accountable for demolitions and displacement, and to instigate protective measures in refugee communities. Policy efforts should prioritize conflict de-escalation, including reaching a sustainable political solution.

## Background

Since Israel assumed the role of occupying power over the occupied Palestinian territories (oPt) in 1967, over 100 Israeli settlements have been established in the West Bank [1,2]. Despite the illegality of these settlements as per Article 49 of the Fourth Geneva Convention [3] and the affirmation of such from the International Court of Justice in 2024 [4], Israeli settlements have continued to expand in the West Bank [5]. The expansion of these settlements has emerged as a significant point of contention, contributing to escalations of violence in the region [6]. Violence takes on the form of altercations between Palestinians and Israelis, but also involves the demolition of Palestinian infrastructure which may result in displacement. Demolitions can be carried out due to the lack of Israeli-issued building permits, which are difficult for Palestinians to obtain [7]. They are also carried out as punitive measures, targeting the families of Palestinians accused of attacks, even when the individuals accused of such attacks are dead or imprisoned [8]. This practice has left thousands homeless and is widely condemned as collective punishment and violation of international law [8].

**Fig 1:**
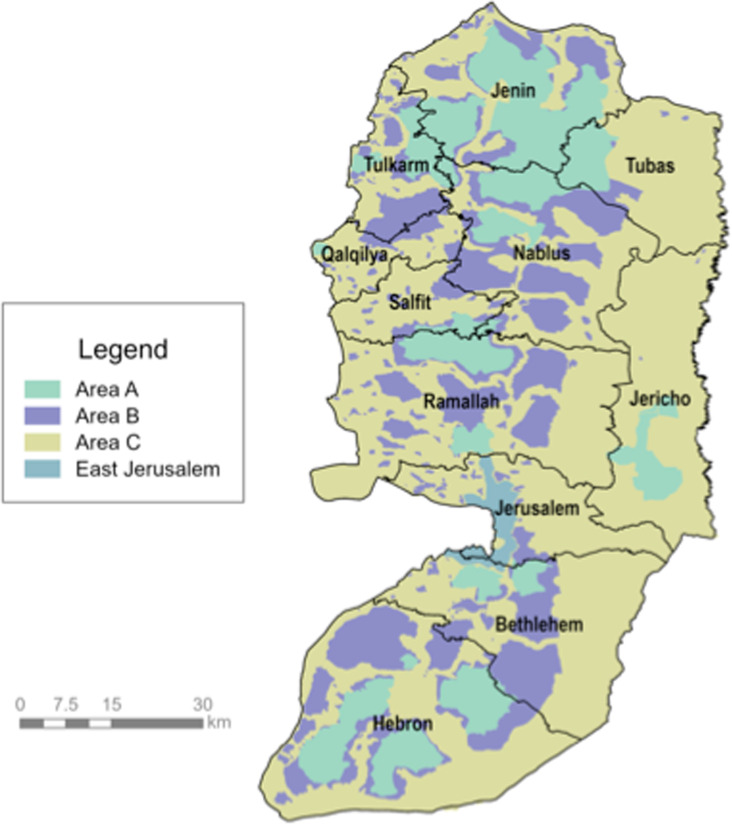
Map of West Bank. Source: Humanitarian Data Exchange [12].

Approximately 63% of the West Bank is considered occupied territory that is controlled by Israeli settlers [2]. This includes settlement jurisdictions which consist of 538,127 dunams, regional councils which control another 2,188,503 dunams, and settlements within Area C which consist of 3,316,787 dunams [9]. The West Bank is divided into distinct areas: A, B, and C, and East Jerusalem (Fig 1), under the Oslo Accords [10]. Area A comprises 18% of the West Bank and is under full Palestinian administrative and security control and is the most densely populated area [10,11]. Area B accounts for about 22% of the West Bank, with the Palestinian Authority overseeing civil affairs and Israel maintaining security control [10,11]. Area C makes up over 60% of the West Bank and is where Palestinian land reserves for development are located; it is under full Israeli military control [10,11].

**Fig 2:**
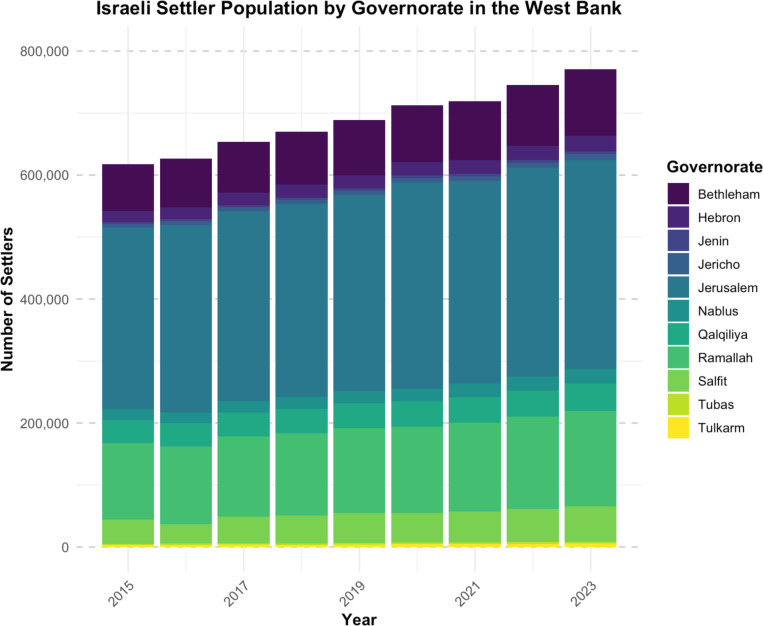
Israeli settler population growth from 2015-2023 in the occupied West Bank. Source: Palestinian Central Bureau of Statistics [14].

Settlement establishment often involves demolition of Palestinian structures and other forms of settler violence leading to civilian displacement [13]. Over the last decade, the Israeli settler population in the West Bank has continued to increase across all governorates (Fig 2).

The interplay between Israeli land occupation and settler aggression has led to escalating hostility and human rights violations within the oPt, resulting in traumatic injuries and deaths for both Israelis and Palestinians, although this disproportionately impacts Palestinians [15]. The resultant humanitarian crisis is exacerbated in the West Bank by fixed movement obstacles that have been deployed by the Israeli military in the form of checkpoints, roadblocks, and earth walls, creating physical barriers that restrict Palestinians’ ability to access appropriate and timely trauma care [16]. Restricted access to trauma care is exemplified in the East Jerusalem neighbourhood of Kafr Aqab, where there are no emergency medical services, including pre-hospital care in the form of ambulances [17]. In cases of emergency, residents must seek care at Jerusalem hospitals [17]. However, as ambulances from Jerusalem do not enter Kafr Aqab, residents must contact ambulance services from the neighbouring city of Ramallah [17]. Patients are then transferred from a Palestinian ambulance to an Israeli one, which must then clear checkpoints to enter Jerusalem [17]. Such delays in trauma care impact patient outcomes and lead to increased morbidity and mortality [18–22].

In recent years, periods of intensified violence have further exacerbated the humanitarian crisis in the West Bank [15,23]. Since the Hamas attacks on Israel on 7 October 2023 and the subsequent onset of the military campaign in Gaza, there has been a sharp rise in settler violence and expansion of settlements in the West Bank, with Israel seizing a record amount of Palestinian land by April 2024 [24]. Of note, as of 25 March 2025 approximately 1,600 Israelis have been killed, most of whom were killed on 7 October 2023 in the Hamas attacks [25], and over 250 hostages were taken [26]. As of the same date, 50,144 Palestinians have been reported killed in Gaza by the Gazan Ministry of Health in the subsequent Israeli military campaign, with research suggesting that this is likely an underestimate [27,28], and over 9,500 Palestinians unlawfully detained [26]. Furthermore, 143,000 Israelis, approximately 1.5% of Israel’s population, were initially displaced following the Hamas attacks [29]. About 1.93 million Palestinians in Gaza, 90% of Gaza’s population has been displaced since the start of the military campaign [30].

UN reports indicate that since 7 October 2023 there have been escalations in Israeli attacks on Palestinian villages in the West Bank resulting in destruction of property and forced displacement of residents [24]. With the recent repeal of Biden-era sanctions against Israeli settlers by the Trump Administration, the limited protections that existed for Palestinians in the West Bank are now gone [31]. As tensions continue to rise, it is crucial to examine how land and military occupation may affect displacement, injuries, and death, further straining an already fragile healthcare system. The aim of this study is to assess how military and land occupation is associated with conflict-related death and injury, and displacement in the West Bank. Such information is needed to inform humanitarian and legal actors such that appropriate interventions can be taken to safeguard Palestinians and their health.

## Methods

### Ethics statement

As the primary data consists of events and their consequent outcomes, with anonymized data regarding casualties, it was considered exempt by the Yale University Institutional Review Board (IRB Protocol ID# 2000039703).

### Study design

This is a cross-sectional observational study that examines conflict-related incidents resulting in casualties (i.e., deaths and injuries), and demolitions in the West Bank and Israel using data from the United Nations Office of Coordination of Humanitarian Affairs (UNOCHA) [7,32], Statista [33], and the Palestinian Central Bureau of Statistics (PCBS) [34]. The analysis covers the period from 1 May 2014 to 30 June 2024.

### Data sources and extraction

This study draws on several publicly available data sources documenting conflict-related incidents resulting in casualties, demolitions, and related metrics in the West Bank and Israel. Anonymized casualty data was extracted from the “OCHA data on casualties” website [32], which provided information on dates, locations, type of incident, perpetrators, weapons used, victims, and outcomes of violent events specifically between Israelis and Palestinians. Data on causalities is regularly collected by UNOCHA field staff and entered into its Protection of Civilians database.

As per UNOCHA protocol, for an incident to be entered onto the database, information including classification of casualties (i.e., if they are civilian, military personnel, part of an armed group, or if their classification is disputed), their demographics (including if they are Palestinian or Israeli), the location of the attack (i.e., Israel, Gaza, or the West Bank) and the context under which the incident occurred must be validated by at least two independent and reliable sources. Incidents resulting in Israeli injuries are an exception to this rule, where information is based on media reports. Only casualties that took place because of confrontations between Palestinians and Israelis in the context of the occupation and conflict are included in the database. Those injured are recorded only if they were physically hurt in a relevant incident and received medical treatment at a clinic or hospital, or by paramedic personnel. Severity of injuries is not provided in the database, nor is it clear if delayed deaths are included. Civilians are defined as persons who are neither members of security forces (including police) nor those who fulfil a combat function within an armed group.

Casualties in the context of the ongoing hostilities in Gaza and Israel, which started on 7 October 2023, have yet to be added to the database as they are awaiting independent verification [32]. However, data on casualties in the West Bank and Israel in other contexts is updated beyond 7 October 2023.

As another potential driver of civilian casualties, demolition data was extracted from the “OCHA data on demolition and displacement in the West Bank” website [7] and includes location of demolitions and displacement caused. Locations are broken down by both community name and area type within the West Bank.

Demographic data was extracted from several sources, including the “Communities in West Bank Index” from the UNOCHA oPt Humanitarian Atlas 2019 [35]. This was used for community-level demographics given that it was the mid-point of the study period and is the only data available from the same source (UNOCHA) as the casualty data for the Palestinian populations included in our study.

Israeli population data by year and ethnicity was extracted from Statista between 2014–2023 [33]. Palestinian and Israeli settler population data by year and governorate from 2014-2023 were extracted from PCBS [34]. 2024 demographic data was not available at the time of analysis.

### Data management

We excluded casualties which occurred in Gaza from the analysis. For the maps of the West Bank, communities located in Israel and nine communities which could not be geolocated were also excluded. Communities which were clearly named “camp,” were categorized as refugee camp communities, while those which did not contain “camp” were assumed to be non-refugee camp communities.

### Data analysis

Descriptive statistics for casualty data, including total number of casualties, distribution of victims and perpetrators, weapon use, communities, and incident type are presented using Excel [36]. Descriptive statistics for demolitions and people displaced, including proportions of those displaced, and monthly demolitions/people displaced were also calculated and, to visualize trends over time, a locally estimated smoothing (LOESS) curve was applied to the monthly data.

To compare the mean conflict-related death and injury rates across the study period, yearly injury and death rates per 100,000 person years were calculated for both Israelis and Palestinians living in the West Bank and Israel. 2024 data is not yet available and was thus not used for this part of the analysis. For the overall analysis, Palestinians living in the West Bank and Israel were combined; the same was done for Israeli settlers in the West Bank and Israelis living in Israel. Because the dataset does not differentiate between Israeli settlers and non-settlers as perpetrators or victims, we assumed that attacks against or perpetrated by Israelis within the West Bank involved settlers. Therefore, we used demographic data on the Israeli settler population for the relevant analyses. The analysis was then stratified to examine these distinct populations in either location using the relevant demographic data as stated in the “Data sources and extraction” section. Rate ratios (and 95% CIs) were then calculated using the yearly death and injury rates for each population by location using the Israeli population as the reference level.

To calculate incidence rate ratios (IRRs), the total number of deaths and injuries were summarized for each community, including camp communities, during the study period. Death and injury rates were then calculated for each community. Poisson regression was used to calculate IRRs between camp and non-camp communities.

Chi-squared analysis was used to examine associations between demolition numbers and area. The number of demolition incidents were grouped by area of the West Bank (Area A, B, C, or East Jerusalem). A chi-square goodness of fit test was then run with the null hypothesis (*H*_*0*_) being that the frequency of demolitions is equal among the four areas. Standardized residuals were then calculated to measure the strength of the differences between observed and expected values, with statistical significance determined as: |R| > 2.

Temporal analysis consisted of aggregation of each outcome by month to create a time-series of the total number of monthly deaths and injuries. An interrupted time-series analysis was then performed to calculate the risk ratio of observed and expected deaths and injuries since October 2023, using a Poisson regression model with a scale parameter to allow for overdispersion in the data. Underlying seasonal patterns in the deaths and injuries data were controlled for using natural cubic splines of time. Similar analysis was conducted for the risk of people displaced since October 2023 for all areas combined as well as separately for those living in Area A. Other areas were not assessed separately due to lack of power.

A GIS shapefile of Areas A, B, C, and East Jerusalem was obtained from Humanitarian Data Exchange [12]. To visualise casualties at the sub-governorate level, a GIS shapefile from PCBS of localities of the West Bank [37] was used as the basis of the maps. To map demolitions at the community level, a GIS shapefile of community coordinates was used [38]. The OCHA dataset contained the names of the communities where injuries and fatalities occurred. For community names that did not match locality names in the PCBS map file, the location of the village was identified by cross-referencing other sources including Google Earth [39] and maps from UNOCHA [40–42] and PeaceNow [9].

Statistical analyses were conducted in R [43] and Stata [44]. Geospatial analysis was done via ESRI ArcGIS Pro software [45].

## Results

### Conflict-related traumatic injuries and deaths

There was a total of 117,014 casualties (i.e., deaths or injuries) recorded in the UNOCHA casualty database as a result of conflict-related incidents during the study period. There were 112,837 (96.4%) Palestinian casualties and 4,177 (3.6%) Israeli casualties. Casualties which occurred in Gaza were excluded from subsequent analyses (n = 43,584). There remained 69,312 total Palestinian causalities (1,092 deaths, 68,220 injuries) and 4,118 Israeli casualties (198 deaths, 3,920 injuries). Of the Palestinian casualties, 69,232 (99.9%) occurred in the West Bank, and 80 (0.1%) occurred in Israel. Of the Israeli casualties, 1,628 (39.5%) occurred in the West Bank and 2,490 (60.5%) occurred in Israel.

**Fig 3:**
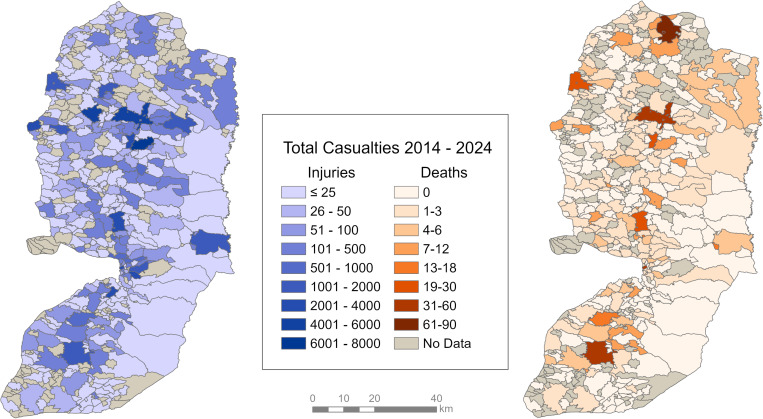
Total injuries and deaths of Palestinians from 2014-2024 in the West Bank by communities. Source: Palestinian Central Bureau of Statistics [37].

Fig 3 shows the spatial distribution of injuries and deaths in the West Bank. The PCBS shapefile contained 585 localities, of which 386 were able to be matched to community names, leaving nearly 200 unmatched or without data. The two communities with the highest total injuries between 2014 and 2024 were in the Nablus Governorate, while the highest number of deaths occurred in two communities in the Jenin Governorate. Of the ten communities with the highest number of injuries, four also ranked among the top ten for fatalities. Refugee camp communities accounted for a total of 4,092 injuries and 275 deaths (5.9% and 25.9% of total Palestinian injuries and deaths in the West Bank).

The average number of monthly deaths during the study period was 8.91 for Palestinians and 1.62 for Israelis. The average monthly injuries for Palestinians and Israelis was 556.90 and 32.00, respectively.

**Fig 4:**
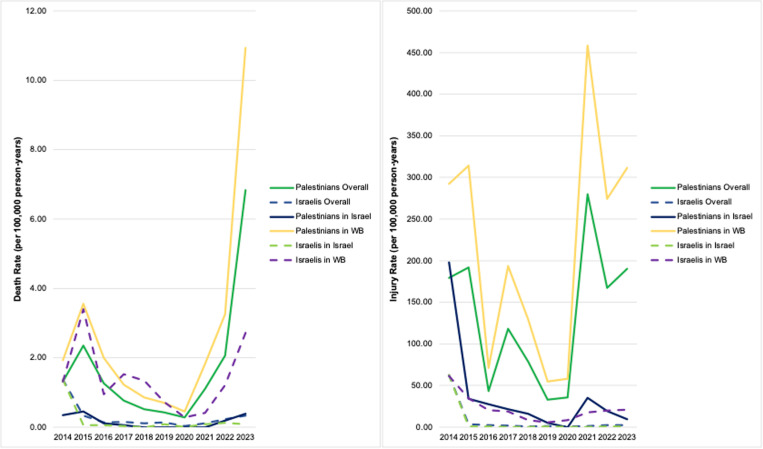
Annual death and injury rates for Palestinians and Israelis, by location.

The mean death and injury rates of Palestinians and Israelis from 1 May 2014 to 30 December 2023 in the West Bank (WB) and Israel are presented in Table 1. Yearly rates are displayed in Fig 4. Across the West Bank and Israel, the death rate was over 5 times greater for Palestinians compared to Israelis and the injury rate was over 16 times greater.

**Table 1:** Mean injury and death rates among Palestinians and Israelis and rate ratios using yearly rate data, stratified by location from 1 May 2014 – 30 December 2023.

Outcome	Population	Mean Rate (per 100,000 person-years)	Rate Ratio (95% CI)	p-value
WB & Israel				
Death	Palestinian	1.69	5.72 (2.38, 13.75)	<0.001
	Israeli	0.30	1.00 (reference)	
Injury	Palestinian	131.80	16.47 (6.86, 39.56)	<0.001
	Israeli	8.00	1.00 (reference)	
West Bank				
Death	Palestinian	2.68	1.92 (0.80, 4.62)	0.43
	Israeli	1.39	1.00 (reference)	
Injury	Palestinian	215.84	9.98 (4.15, 23.98)	<0.001
	Israeli	21.62	1.00 (reference)	
Israel				
Death	Palestinian	0.16	0.81 (0.34, 1.95)	0.64
	Israeli	0.19	1.00 (reference)	
Injury	Palestinian	36.7	5.47 (2.28, 13.15)	<0.001
	Israeli	6.70	1.00 (reference)	

Notably, 69,163 (99.8%) of Palestinian casualties were unarmed civilians, while 1,258 (30.5%) of Israeli casualties were unarmed civilians. 790 (19.2%) of Israeli causalities were military or non-military security forces, however, 2,069 (50.2%) of Israeli casualties were classified as “unknown” in terms of their military versus civilian status. The majority of both Palestinian and Israeli casualties were male: 61,819 (97.9%), and 3,882 (94.3%) respectively.

During the study period, death and injury rates were significantly higher in refugee camps compared to non-refugee camp communities. The incidence rate ratio (IRR) for camp vs. non-camp deaths was 7.91 (95% CI: 5.26–11.89, *p* < 0.0001) and 1.20 (95% CI: 1.14–1.27, *p* < 0.0001) for injuries.

### Typology of attacks

**Fig 5:**
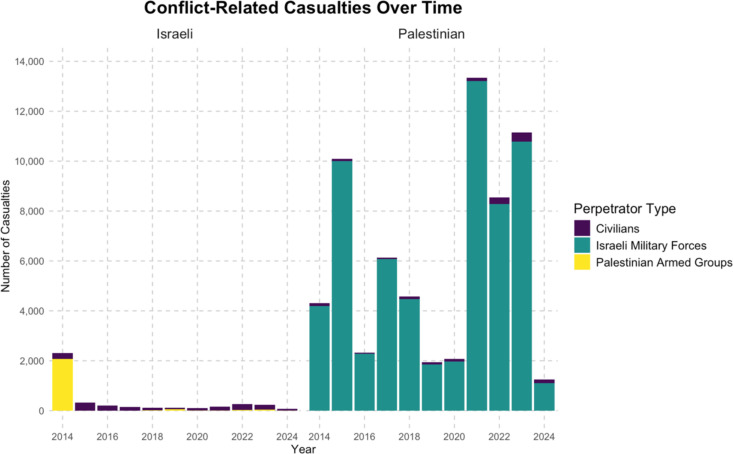
Conflict-related casualties, Israelis (left) and Palestinians (right), and their associated perpetrators, during the study period in the West Bank and Israel.

The majority of the attacks against Palestinians were perpetrated by Israeli military forces during the study period, accounting for 64,218 (92.7%) of the total casualties. Attacks by Israeli civilians made up 1,512 (2.2%) of incidents. The remaining perpetrators were categorized as “disputed.” The majority of attacks against Israelis were perpetrated by Palestinian armed groups 2,251 (54.7%); 2,033 (90.3%) of attacks by Palestinian armed groups occurred in July and August 2014. The remaining 1,849 (44.9%) of attacks against Israelis were perpetrated by Palestinian civilians. Fig 5 shows the evolution of casualties and perpetrators over time. 2024 had the highest percentage of Israeli civilian perpetrated violence against Palestinians at 11.4%. Palestinian and Israeli casualties on 7 October 2023 are not included within this figure as they had not been added to the UNOCHA dataset at the time of data analysis due to ongoing independent verifciation.

Weapons used against Palestinians are shown in Table 2. Weapons used against Israelis were not reported in the data set.

**Table 2:** Weapons used in conflict between Israelis and Palestinians during the study period.

Recorded Weapon	Used Against Palestinians N (%)
Tear gas (inhalation)	41,057(56.4%)
Rubber bullets	14,118 (19.4%)
Live ammunition	7, 489 (10.3%)
Other	6,061 (8.3%)
Physical assault	3,541 (4.9%)
Tear gas (hit)	582 (0.8%)
Set on fire	5 (0.007%)
Total	72,853 (100%)

The use of crowd-control weapons such as tear gas and rubber bullets against Palestinians is noteworthy since 34,182 (49.3%) of all recorded incidents were demonstrations. Conversely 2,071 (50.3%) of incidents against Israelis were related to major hostilities, namely the 2014 Gaza War.

### Demolition and displacement analysis

There were a total number of 2,740 demolition incidents recorded in the UNOCHA demolition database during the study period. Of these, 127 (4.6%) occurred in Area A, 61 (2.2%) in Area B, 1,443 (52.7%) in Area C, and 1,109 (40.5%) in East Jerusalem. Table 3 shows the average monthly demolitions and displacements for each area.

**Table 3:** Average monthly demolished structures and displaced Palestinians in the West Bank during the study period.

	Area A	Area B	Area C	East Jerusalem
Average monthly demolitions (structures per month)	3.42	0.76	39.12	13.71
Average monthly displacement (people per month)	16.73	2.31	43.16	25.83

**Fig 6:**
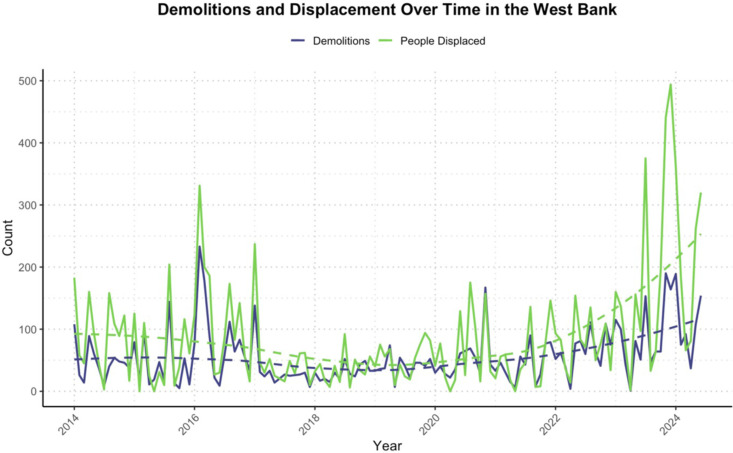
Monthly structures demolished and people displaced with smoothed trends (dashed lines) during the study period. Smoothed trends are based on LOESS functions.

Structures demolished and people displaced have generally been increasing over time (Fig 6). During the study period there was a total of 10,778 people displaced.

**Fig 7:**
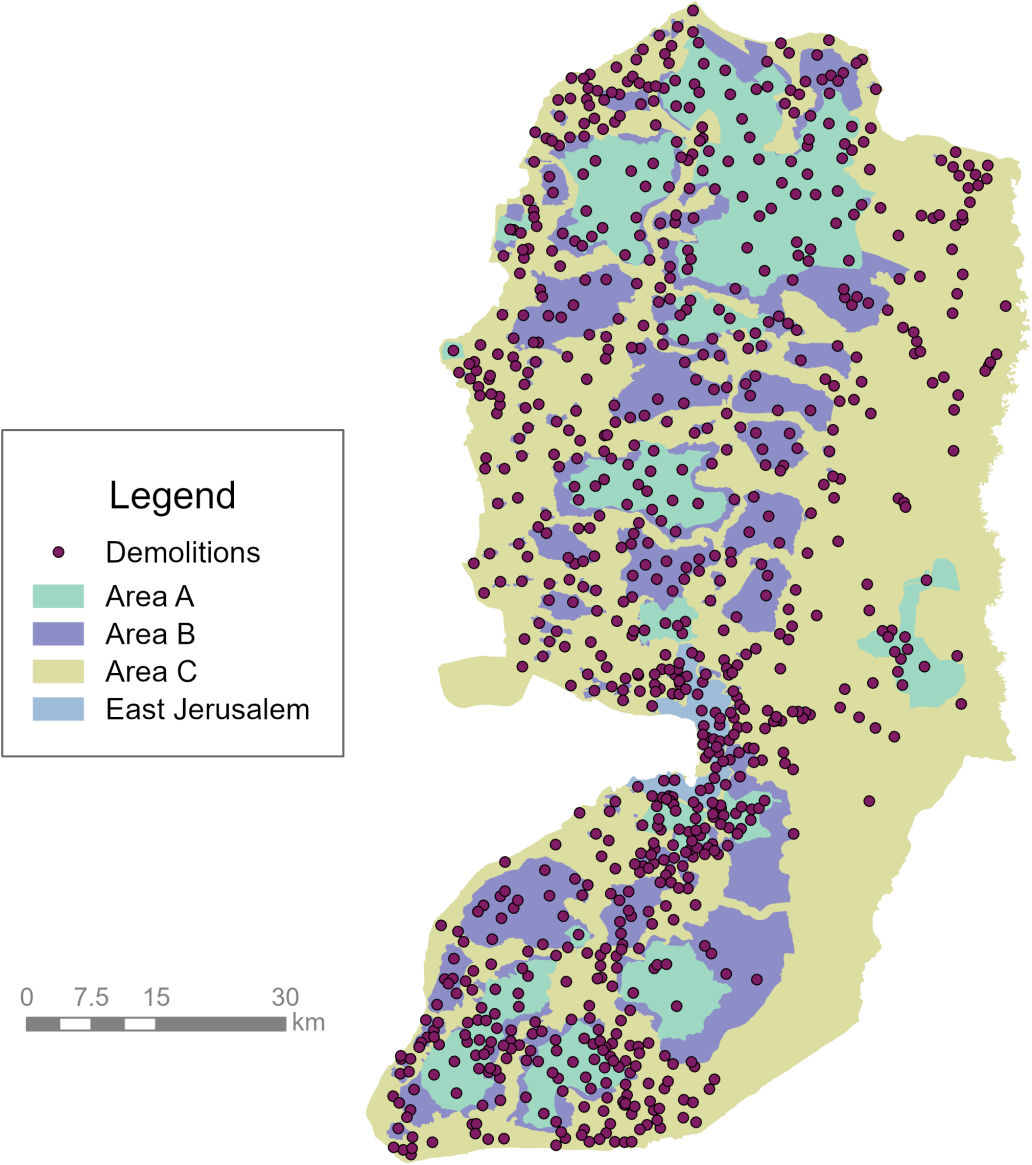
Locations of demolished structures in West Bank communities during the study period. Source: Humanitarian Data Exchange [12,38].

Fig 7 shows the spatial distribution of demolished structures in the West Bank. A total of 403 out of 409 community names from the demolition data were matched to the coordinates of 850 West Bank communities obtained from the Humanitarian Data Exchange [38]. The highest concentration of demolished structures occurred in East Jerusalem, with the community of Jabal al-Mukkabir alone accounting for 311 demolished structures during the study period.

Demolition patterns across the West Bank were not uniform, with significant variation observed between areas (χ² = 2124.2, *p* < 0.0001). Area C and East Jerusalem had significantly higher observed counts than expected (R = 29.63 and 16.27, respectively), while Areas A and B had significantly lower observed counts than expected (R = -21.70 and -24.19, respectively).

### Temporal analysis

Analysis of daily injury data revealed that 38.8% of injuries occurred on Fridays, indicating a notable weekly pattern. The most dangerous period identified was 10–18 May 2021, which recorded the highest number of injuries.

**Fig 8:**
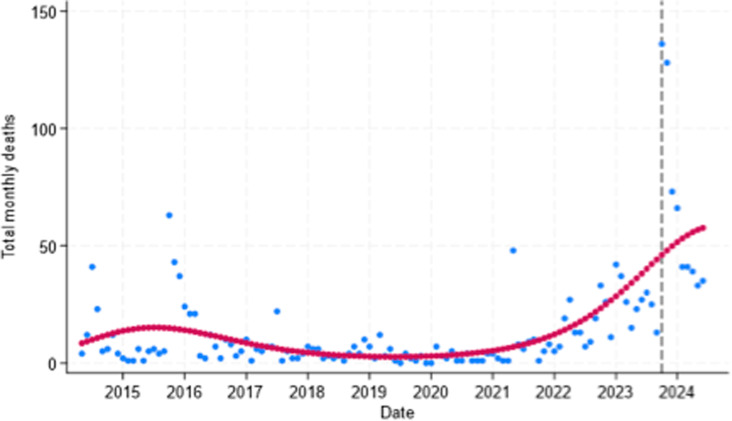
Total monthly deaths of Palestinians in the West Bank May 2014 to June 2024. Blue dots = observed deaths; red dots = predicted deaths. Dashed black line = Oct 2023.

Fig 8 shows the total number of deaths by month in the West Bank. Deaths saw a sharp rise since 7 October 2023. After controlling for underlying seasonal patterns in the monthly death data, the risk of death was over 25% higher than expected in the period since October 2023: RR = 1.25 (95% CI: 1.15, 1.36; *p* < 0.0001). The number of injuries was also higher than expected since October 2023: RR = 1.27 (95% CI: 1.24, 1.31; *p* < 0.0001).

**Fig 9:**
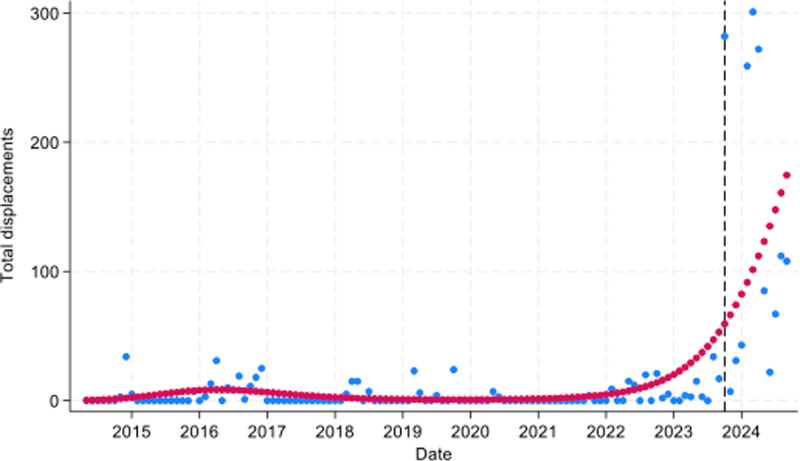
Total monthly Palestinians displaced in Area A during May 2014 to June 2024. Blue dots = observed peopled displaced; red dots = predicted people displaced. Dashed black line = Oct 2023.

Displacements also saw a sharp rise after October 2023. Across all areas, the risk of displacement was 17% higher than expected since October 2023: RR = 1.17 (95% CI: 1.12, 1.21; *p* < 0.0001). Those living in Area A saw over a 15% increase in displacement compared to expected levels since October 2023: RR = 1.15 (95% CI: 1.16, 1.22; *p* < 0.0001) (Fig 9). Structures demolished have seen a general upward trend (Fig 6) and were 11% higher than expected since October 2023: RR = 1.11 (95% CI 1.04, 1.18; *p =* 0.002).

## Discussion

The findings of our study confirm a significant disparity in rates of conflict-related traumatic injuries and deaths between Palestinians and Israelis in the occupied West Bank and Israel, whereby there is a disproportionate impact of violence on Palestinian communities.

The widespread use of crowd-control weapons against Palestinians, particularly tear gas and rubber bullets align with the fact that nearly half of the recorded incidents occurred during demonstrations. Similar dynamics of state violence against civil demonstrations are seen in other contexts, including the United States and other parts of the Middle East [46–48]. Within the Palestinian context, many demonstrations are held with regards to settlement expansion [49–51]. Thus, there is a possible impact of settlement expansion on exposure of violence in Palestinian communities.

The clustering of injuries on Fridays suggests a possible link between religious gatherings and increased exposure to violence. This pattern aligns with existing research, which describes a possible relationship between mass gatherings, including religious gatherings, and a heightened risk of injuries [52,53].

Most attacks against Palestinians were perpetrated by Israeli military forces, indicating the dominance of state-led violence in shaping the traumatic injury landscape for Palestinians. Attacks against Israelis were primarily carried out by Palestinian armed groups and civilians, reflecting a mix of organized and individual acts of violence rather than centralized military action against Israelis.

The overwhelming majority of Palestinian casualties were unarmed civilians, while a notable proportion of Israeli casualties included military forces. However, a substantial proportion of Israeli causalities were classified as “unknown,” so the true scale of military versus civilian casualties remains unclear. Nevertheless, the results raise concerns about the targeting of Palestinian civilians. The gender distribution of casualties also indicates that males bear the brunt of conflict-related violence on both sides.

Residency in refugee camps was identified as a risk factor for conflict-related traumatic injury outcomes for Palestinians, with significantly elevated rates of both injury and death compared to non-camp communities. This heightened vulnerability aligns with the literature regarding refugee camps and injury in other settings, such as Lebanon, Syria, Kenya, and other countries within the African continent [54–58]. This may be due to their dense population, limited access to protective infrastructure and restricted mobility. A spatial analysis of 1,543 refugee camps across Africa found that conflict events frequently occurred near these camps, a pattern that may parallel dynamics in the West Bank [58].

The analysis suggests an interconnection between violence against Palestinians in Gaza and the West Bank. The period from 10-18 May 2021 marked the most dangerous period during the study for Palestinians in the West Bank, corresponding with the 2021 Gaza War. The increased risk of deaths and displacement of Palestinians in the West Bank since 7 October 2023 underscores the intensification of hostilities in both regions, and the urgent need for interventions to also protect civilians in the West Bank. The significant role of Israeli civilians and settlers in violent incidents in 2024 may signal shifting patterns of violence, and the need for measures such as reinstating and broadening Biden-era sanctions against Israeli settlers, which were recently removed by the Trump administration [31].

Demolitions were found to be disproportionately concentrated in certain regions, with Area C and East Jerusalem experiencing significantly higher observed demolitions. This aligns with the political context of both regions, which are both under full Israeli military occupation.

Conversely, Areas A and B show significantly lower observed demolitions which are under Palestinian Authority control. Despite this, demolitions continue to occur in all areas, and especially in the period after October 2023, causing concerns for encroaching Israeli control and further displacement of Palestinians. Thus, there should also be efforts to hold the Israeli government accountable for continued demolitions and consequent forced displacement of Palestinians, with heightened attention to Area A, which has experienced a significant increase of displacement since October 2023.

Future efforts should focus on deconfliction activities, including the extension of ceasefire agreements beyond Gaza to include the West Bank. Literature has shown that full military withdrawal, international monitoring, and political settlements are strongly associated with lasting ceasefires [59,60]. The strongest among these factors is a political settlement: in fact, it appears many ceasefires between Israel and Palestine have failed due to the lack of an imposed upon or agreed upon political solution [59]. International and humanitarian actors should also advocate for the implementation of protective measures in refugee communities where Palestinians experience significantly higher injury and death rates.

Strengthening emergency care systems to improve trauma response and ensure access to life-saving care should also be a priority to mitigate traumatic injury outcomes due to ongoing hostilities in the West Bank [61]. This includes adequate emergency and trauma training, and ensuring the safe, timely delivery of medical services starting with pre-hospital care. In lieu of a current political solution, this measure may safeguard Palestinian lives until a political settlement is reached.

### Limitations

A key consideration is the observational nature of the study design which prohibits strong determination of causality. Other limitations are that minor injuries are not recorded within the database leading to underreporting, and Palestinian casualties are only recorded if there is two-source verification, whereas this is not the case for Israeli casualties. This may lead to underestimation of Palestinian injury rates. Furthermore, given that two-source verification is only used for Palestinian casualties, there is a risk of misclassification bias for Israeli casualties, including those that may be Palestinian citizens of Israel. It is also unclear if delayed deaths due to conflict-related injury are updated within the database. Furthermore, casualties related to the hostilities as of 7 October 2023 are not included within the database, which may lead to underestimation of both Palestinian and Israeli death and injury rates.

Weapon data was not available for those used in incidents against Israelis, limiting comparison between the two groups. The lack of detailed casualty data prevents a comprehensive demographic analysis, including age-specific trends. An analysis looking at area designations would have permitted more robust examination of the effects of military occupation on traumatic injury outcomes, but this was not available for the casualty dataset.

Additionally, geospatial analysis could be enhanced by incorporating coordinate data for both demolitions and casualties, enabling the use of more robust statistical methods such as hotspot analysis. However, coordinates were unavailable in both OCHA datasets on demolitions and casualties. Furthermore, there were many communities in the West Bank for which data was missing; it is unclear if this is due to lack of reporting or if no deaths or injuries truly occurred in these communities during the study period. This may result in underestimation of death and injury rates, or of over or underestimation of the IRR between refugee and non-refugee camp communities.

Assessment of spatial patterns of demolitions could have been improved by defining expected frequencies based on historical patterns of demolition. However, demolition data only began being recorded in 2009, and earlier data may not accurately reflect true demolition trends. Additionally, expected values could have been calculated based on relative land areas or proportions based on building numbers in each area, but this approach would overlook the geopolitical context of each area and the total building numbers per area were not available. While the current analysis is simplified, it provides a baseline to assess whether some areas experience more demolitions than others.

## Conclusion

This analysis provides insight into patterns of traumatic health outcomes, demolition, and displacement, in the occupied West Bank. It confirms the disparity in conflict-related traumatic injury and death between Israelis and Palestinians, and suggests that areas that are under stricter Israeli control (i.e., Area C and East Jerusalem) may have higher incidences of demolition and displacement. The period after October 2023 has also seen higher rates of injury, death, and displacement amongst Palestinians in the West Bank.

Deconfliction methods may mitigate conflict-related violence amongst Palestinians and Israelis. Strengthening emergency care and removing barriers to receiving emergency care may improve traumatic injury outcomes amongst Palestinians. Further studies examining the long-term health and psychological consequences of occupation on the health of those currently residing within the oPt and Israel are needed. This would further assist humanitarian and governmental organisations meet the needs of each given population.
